# Uretero-Fallopian Fistula after excision of ovarian cyst

**DOI:** 10.12669/pjms.41.8.11240

**Published:** 2025-08

**Authors:** Sohail Hassan, Kiren Khurshid Malik, Sidra Afzal, Kamran Ashraf

**Affiliations:** 1 Sohail Hassan Professor of Urology and Consultant Urologist, Social Security Teaching Hospital, Lahore, Pakistan. University College of Medicine and Dentistry, Lahore, Pakistan; 2 Kiren Khurshid Malik Professor of Gynae and Obstetrics, Lady Wallingdon Hospital, King Edward Medical University, Lahore, Pakistan; 3 Sidra Afzal Assistant. Professor and Consultant Radiologist, Social Security Teaching Hospital, Lahore, Pakistan. University College of Medicine and Dentistry, Lahore, Pakistan; 4 Kamran Ashraf Radiologist, Social Security Teaching Hospital, Lahore, Pakistan. University College of Medicine and Dentistry, Lahore, Pakistan

**Keywords:** Salpingoureteric fistula, Ureterofallopian fistula, Urogenital fistula

## Abstract

Injuries to the urinary tract are regrettable consequences of pelvic surgery. These often pertain to the bladder. The prevalence of iatrogenic ureteral injuries varies between 0.05% and 30%. Although certain lesions are identified intraoperatively, the majority remain undetected and manifest thereafter. Ureteral fistulas typically affect the vagina and, less commonly, the uterus. Uretero-fallopian fistulas are exceedingly uncommon. We present a case of uretero-fallopian fistula that emerged following the resection of an ovarian cyst.

## INTRODUCTION

Urogenital fistula is a distressing and debilitating illness for women, resulting in their social isolation. It is believed that between 30,000 to 130,000 new cases are recorded globally each year.^1^ Approximately 3,500 new instances of urogenital fistula are documented annually in Pakistan. The majority are vesicovaginal fistulae; nevertheless, a significant proportion are ureterovaginal fistulae. The majority of ureterovaginal fistulae are attributable to iatrogenic injury.^2^ Approximately 73% of cases arise as iatrogenic injuries after gynecological surgeries,14% during general surgical procedures, and 13% during urological surgeries.^3^

The early diagnosis of ureteric injuries is crucial for their care and outcome; however, this is not always feasible until patients exhibit relevant signs and symptoms post-surgery. These symptoms may include discomfort or sepsis, perhaps resulting in renal failure or urinary incontinence.^4^ A fistula between the fallopian tube and ureter is an exceedingly unusual complication that arises following pelvic procedures involving the ureter, fallopian tube, or ovaries, as demonstrated in this case. Such fistulae can lead to significant problems such as hydronephrosis and infections, and their repair is a considerable surgical challenge.^5^ Upon reviewing the literature, every documented uretero-fallopian fistula arose following significant surgical interventions such as rectal cancer operations, open ureterolithotomy, or endometrioma excision. One case is documented following hysteroscopy and fallopian tube embolization. No instances have been documented in which the excision of an ovarian cyst resulted in the formation of a uretero-fallopian fistula.

## CASE PRESENTATION

A 32 years old female patient appeared in the outpatient department with a complaint of urinary incontinence for the past eight months. She remained damp and was capable of urinating independently. She additionally reported periodic pain in the left lumbar region. The pain was minor, intermittent, and subsided following the administration of NSAIDs. Her cystoscopic examination was unremarkable, despite urine emanating from the uterus. Eight months ago, she underwent a laparotomy for the excision of a left ovarian cyst. Two weeks’ post-surgery, she began experiencing urinary leakage. The histopathological examination of the ovarian cyst wall revealed loose fibrovascular connective tissue, variably bordered with hemorrhagic material, granulation tissue, fibrin, and pigmented macrophages. No functional endometrial-type glands were seen. No evidence of cancer was present. The diagnosis was identified as degenerative and atrophic endometriosis. An ultrasound was recommended one-month post-surgery, which indicated the presence of around 2ml of free fluid at the incision site. She received conservative management. A Micturating Cystourethrogram (MCUG) was performed, revealing normal results with no urinary leakage from the bladder. The patient was subsequently referred to the urology department for additional care. A CT IVU was performed, revealing left-sided hydronephrosis with a dilated ureter extending to the distal ureter at the S2 level, where it communicated with a cystic lesion measuring 49x36 mm.

**Fig.1:**
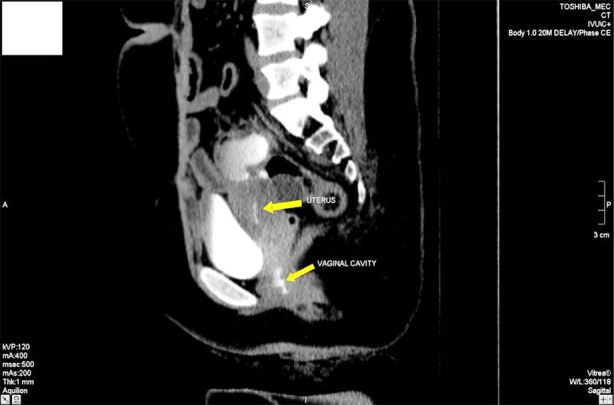
Showing contrast in uterus and vaginal cavity. There is urinoma communicating between ureter and fallopian tube.

During the delayed excretory phase, this lesion was enhanced with contrast and was in communication with the distal ureter. Extrinsic compression was observed on the neighboring sigmoid colon; however, no intraluminal extension was detected. The neighboring dilated fallopian tube was similarly filled with contrast material. Contrast was also observed in the uterine cavity and vagina. As seen in Fig.1. Based on these radiological findings, a diagnosis of “Uretero-Fallopian Fistula” was established. The patient also had urinary incontinence.

The cystoscopy revealed no fistula tract and a normally sized urine bladder. Retroperitoneal exploration was performed by a grid-iron incision on the left side, which was subsequently extended toward the urinary bladder. The retroperitoneal space was examined. There were strong adhesions. Adhesion lysis performed meticulously to delineate the anatomical structures. The fallopian tube was meticulously identified and dissected. The ureter was excised from the cystic lesion (urinoma) and removed together with its surrounding walls. The urinary bladder was mobilized, and the left ureter was re-implanted over a Double-J stent.

**Fig.2:**
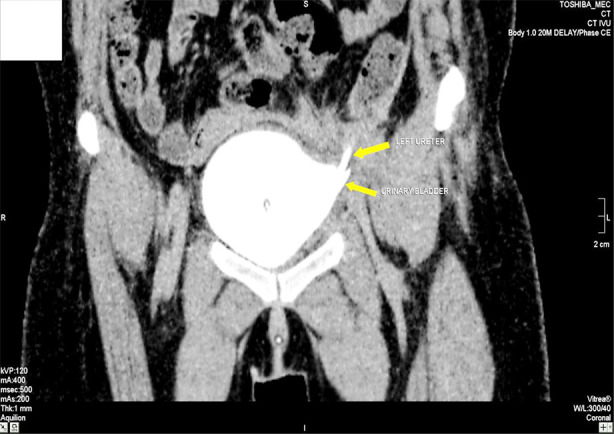
Post-operative scan showing urinary bladder and reimplanted ureter.

A drain was implanted adjacent to the urinary bladder, and the wound was closed in layers. A broad Foley catheter was also inserted. Following a three-day period, the drain was extracted, and after two weeks, the Foley catheter was withdrawn. CT-IVU was repeated at six weeks’ post-surgery. No evidence of fistulous communication or collection was observed in the operating area. As seen in Fig.2. Follow-up was recommended biweekly for a duration of six months. The patient-maintained continence and expressed satisfaction with her health, reporting no complaints.

## DISCUSSION

Numerous instances of fistulae between the bowel and fallopian tubes have been documented in literature, while there are only a limited number of cases involving fistulae between the ureter and fallopian tube. The majority of these cases stemmed from iatrogenic injuries during rectal cancer surgeries and laser procedures for endometriosis. A case was documented during laparoscopic rectal cancer surgery, while another instance involved hysteroscopy and fallopian tube embolization.^5^

In 1988, Christmas and Badenoch described a case of 59 years old, diabetic, female patient who reported with pain in the right lumbar region. She had previous history of recurrent urinary tract infections and bilateral renal calculi. On radiological investigations there was presence of gas in bony pelvis, ureter and kidney and gross hydronephrosis with loss of renal parenchyma. On Cystoscopy there was oedematous Right ureteric orifice and complete obstruction 1cm above ureteric orifice. On Laparotomy, the right ureter was fixed with Fallopian Tube and surrounded by abscess. The right nephroureterectomy and Right salpingectomy was performed. The histopathology revealed the presence of an inflammatory mass connecting the ureter and fallopian tube.^6^

In 1992, Huang et al, reported another case involving a female patient who underwent laser surgery for endometriosis.^6^ The CT scan indicated the presence of a uretero-fallopian fistula. In 1993, Brasils and Stephens documented an additional instance of Uretero-Fallopian Fistula following an uncommon complication of ureterolithotomy. The patient presented with urinary incontinence.^7^

Steckel et al, documented a case of uretero-fallopian fistula that developed three weeks subsequent to the laparoscopic fulguration of endometriosis.^8^ The patient appeared with stomach pain and urinary incontinence. Crochet et al, have documented a case of uretero-fallopian fistula following endometriosis surgery.^9^ Recently, Zhang Y et al. reported an additional instance of Uterine Fibroid Formation (UFF) following hysteroscopy and embolization of the fallopian tube.^5^

### Author’s Contribution:

**SH:** Performed surgery, Acquisition of data and design of work.

**KKM:** Assisted in surgery, Drafting and critical review.

**SA:** Radiologist, Collection and interpretation of Data and accountable for the accuracy of the study.

**KA:** Secondary Radiologist, Collection and interpretation of Data.

All authors have read and approved the final version of the manuscript.
